# Supplementary material for: Genome-Wide Selective Signature Analysis Revealed Insecticide Resistance Mechanisms in Cydia pomonella
Source: Insects. 2021 Dec 21;13(1):2. doi: 10.3390/insects13010002 (PMC8781923; doi:10.3390/insects13010002)
Supplement: Supplementary file 1 [file insects-13-00002-s001.zip › Figures S1-S6.pdf]

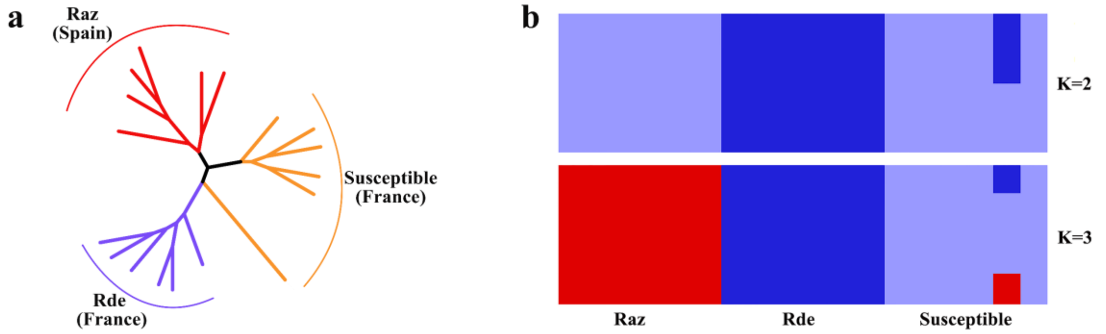

Figure S1. (a) Unrooted phylogenetic tree of *Cydia pomonella*. (b) Population structure plots with K=2, 3. The *y*-axis quantifies the proportion of the individual's genome from inferred ancestral populations, and *x*-axis shows the different populations. The Rde and susceptible strains were from south-eastern France, and the Raz strain from Lerida, Spain.

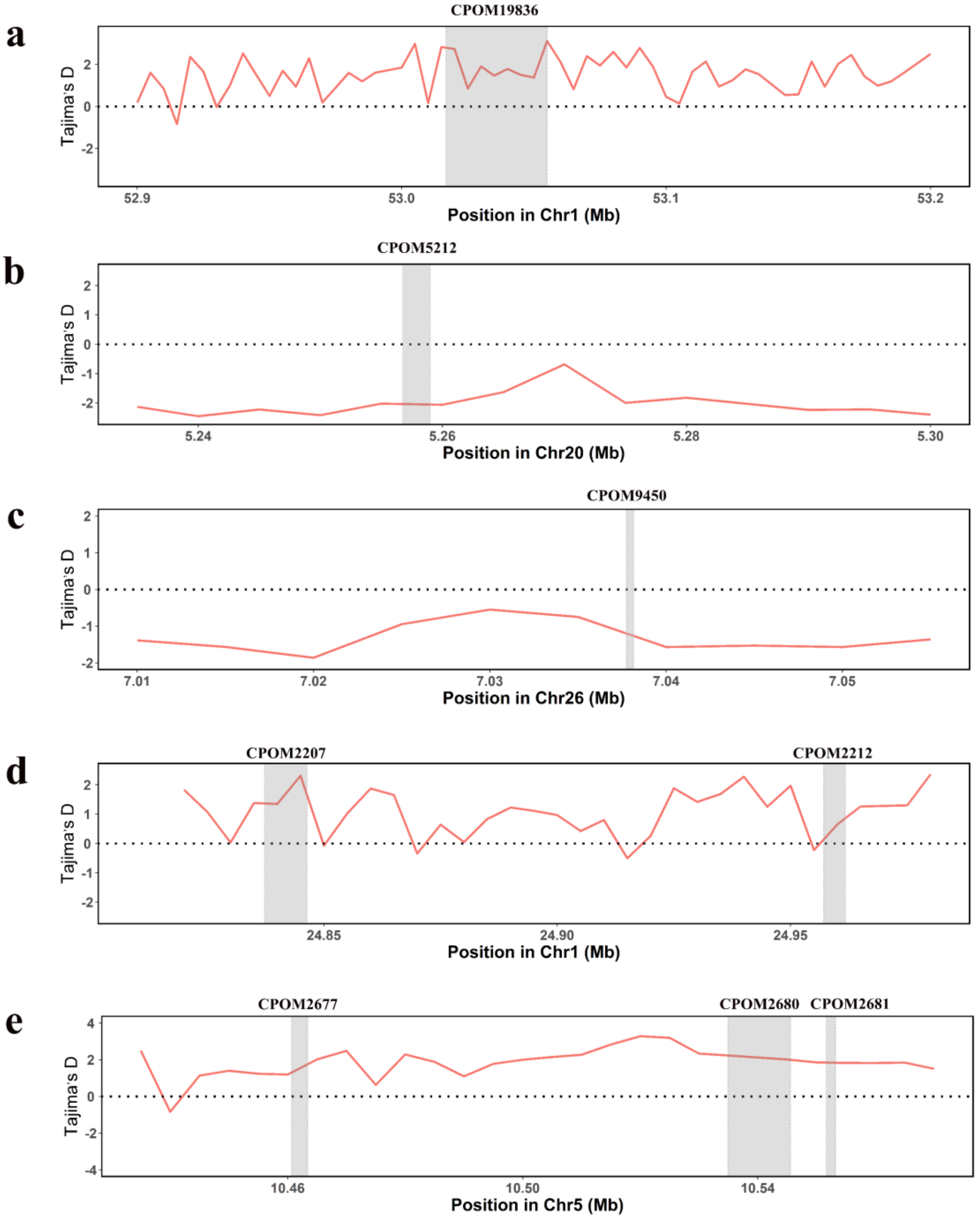

Figure S2. Tajima's D of the eight PSGs (listed in Table 1) in resistant Raz and Rde strains of *Cydia pomonella* (n=12)). (a) CPOM19836: transient receptor potential cation channel subfamily A member 1. (b) CPOM05212: cytochrome P450 6B2. (c) CPOM09450: cytochrome P450 307a1. (d) CPOM02207: 5-hydroxytryptamine receptor 2A; CPOM02212: acetylcholinesterase. (e) CPOM02680: cuticle protein 8; CPOM02681: cuticle protein 19; CPOM02677: cuticle protein 19. The red lines show the Tajima's D of genome area around PSGs. The *x*-axis represents the location in chromosomes (Mb), and grey area shows locations of PSGs.

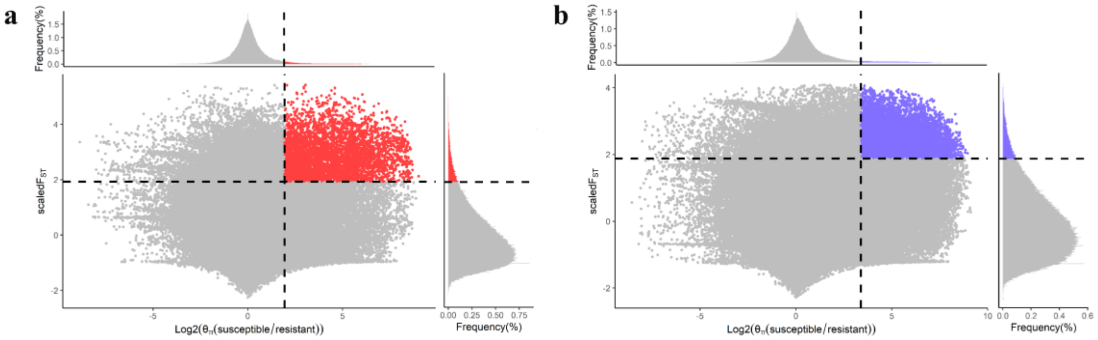

Figure S3. Distribution of F_{ST} and θπ ratio (θπ(susceptible/resistant)) in (a) Raz and (b) Rde strains of *Cydia pomonella*, respectively, the *x*- and *y*- axes with associated frequency plots. The top-right corner where red (or purple) points are located represents genome regions under selection pressure for azinphos-methyl and deltamethrin. The horizontal and vertical gray dashed lines represent the top 5% value of F_{ST} and $\log_2$(θπ(susceptible/resistant)).

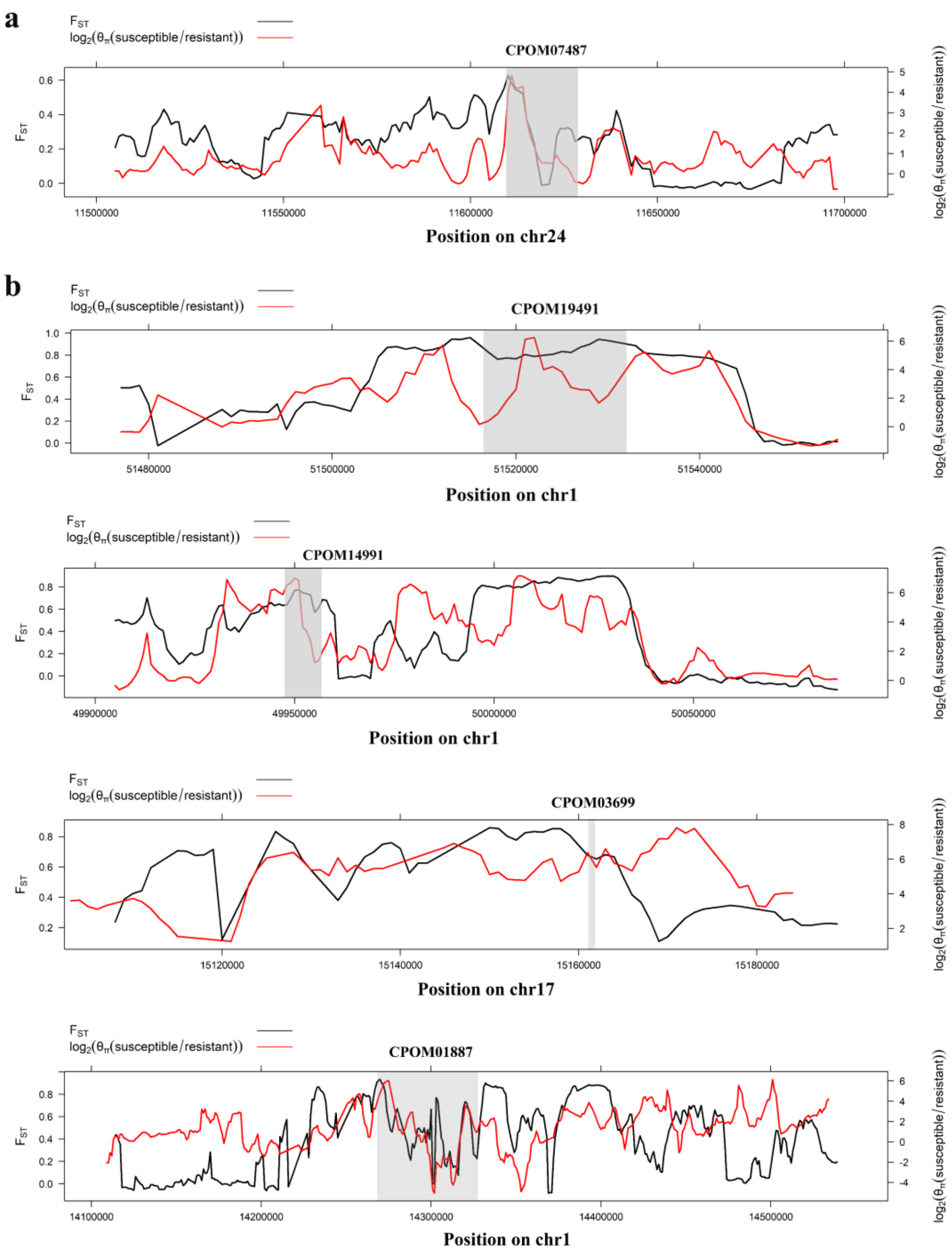

Figure S4. F_{ST} and θπ ratio of the PSGs in (a) Raz and (b) Rde strains of *Cydia pomonella*. The red and black lines show the F_{ST} and θπ(susceptible/resistant) of genome area around *glycine receptor* and *glutamate receptor*. The *x*-axis represents the location in chromosomes (bp), and the grey area shows the location of *glycine receptor* and *glutamate receptor*. CPOM14991: glutamate receptor 1; CPOM19491: chitinase-like protein; CPOM07487: glycine receptor subunitalpha-2; CPOM03699:5-hydroxytryptamine receptor 2A; CPOM01887: gamma-aminobutyric acid type B receptor subunit 1.

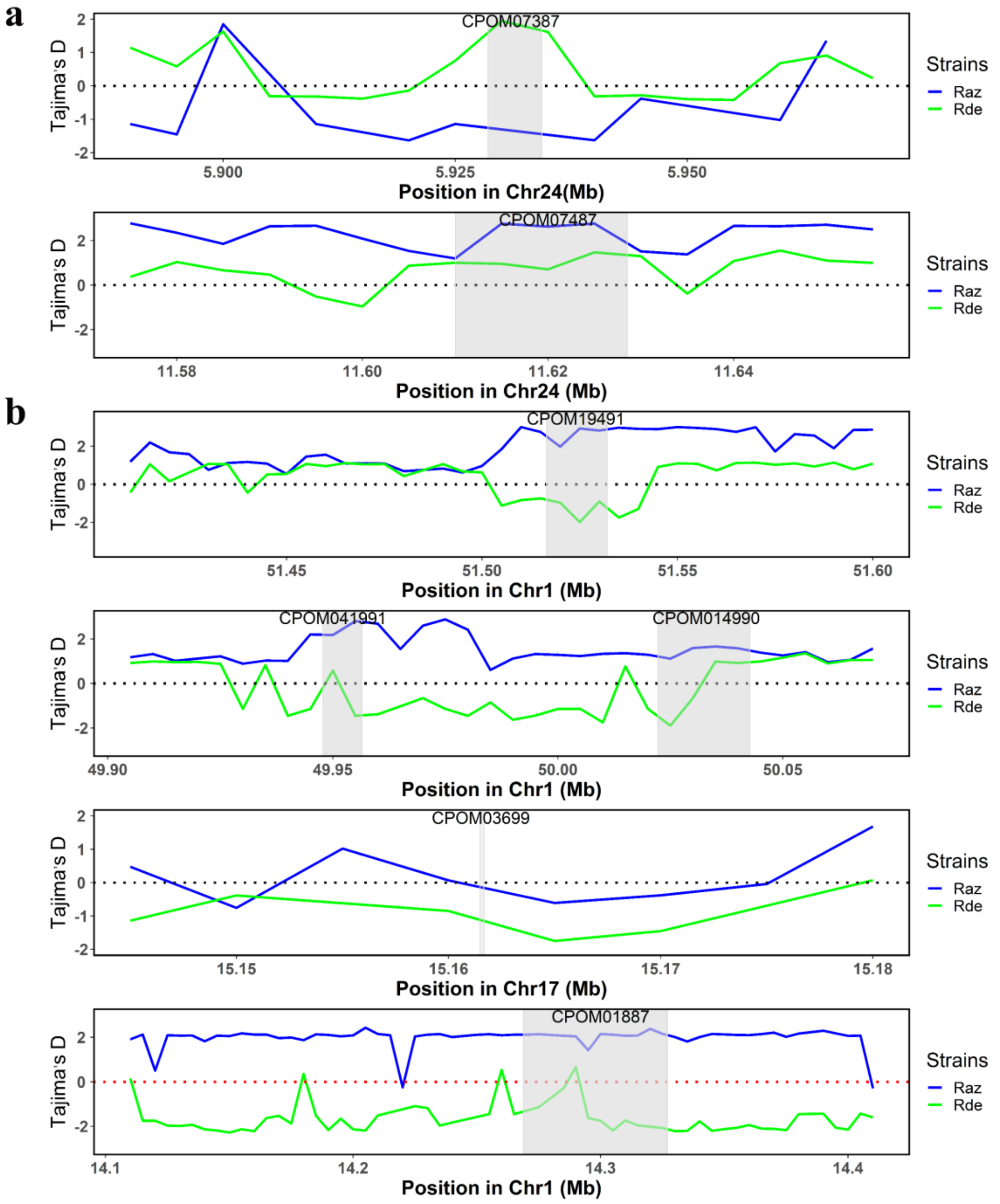

Figure S5. Tajima's D of the PSGs in (a) Raz and (b) Rde strains of *Cydia pomonella*. The blue and green lines show the Tajima's D of genome area around PSGs. The *x*-axis represents the location in chromosomes (Mb), and the grey area shows the location of PSGs. CPOM14991: glutamate receptor 1; CPOM19491: chitinase-like protein; CPOM07487: glycine receptor subunit alpha-2; CPOM03699: 5-hydroxytryptamine receptor 2A; CPOM01887: gamma-aminobutyric acid type B receptor sub-unit 1; CPOM07387: Glycine receptor subunit alpha-2; CPOM14990: Glutamate receptor 1.

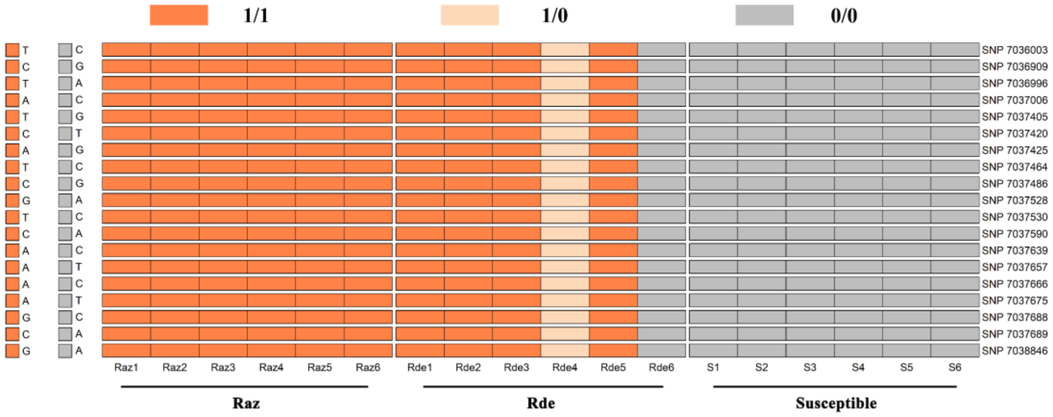

Figure S6. Genotype variance of 11 SNPs of the *CYP307a1* gene (geneID: CPOM09450) between Raz, Rde and susceptible strains of *C. pomonella*. SNPs were named according to their position on the chromosome. The orange grids represent the homozygous SNPs (1/1). The light orange grids represent the heterozygous SNPs (0/1).
